# The effect of interrupted anti-retroviral treatment on the reconstitution of memory and naive T cells during tuberculosis treatment in HIV patients with active pulmonary tuberculosis

**DOI:** 10.4314/ahs.v17i4.2

**Published:** 2017-12

**Authors:** Sophie Nalukwago, Christina L Lancioni, Joy Baseke Oketcho, Dave H e Canaday, W Henry Boom, Lonzy Ojok, Harriet Mayanja-Kizza

**Affiliations:** 1 Joint Clinical Research Centre, Kampala, Uganda; 2 Department of Paediatric infectious diseases, Oregon Health Sciences University, Portland Oregon; 3 Division of Infectious Diseases, Case Western Reserve University; 4 Getriatric Research Center Clinical Core, Louis Stoves Cleveland VA Medicine Center; 5 Department of pathology, Makerere University College of Veterinary Medicine, Animal resources and biosecurity, Kampala, Uganda; 6 School of Medicine, Makerere University College of Health Sciences, Mulago Hospital, Kampala, Uganda

**Keywords:** Interrupted anti-retroviral treatment, memory and naive T cells, HIV patients, active pulmonary tuberculosis

## Abstract

**Background:**

The reconstitution of cellular immune components contributes to clinical outcome of HIV and *Mycobacterium tuberculosis* (MTB) infection. Interruption of anti-retroviral therapy (ART) could lead to perturbations in reconstitution of T cells in HIV/ tuberculosis (TB) patients.

**Objectives:**

To ascertain the effect of interrupted ART on reconstitution of CD4^+^ and CD8^+^ T sub-sets in TB patients.

**Methods:**

Participants with HIV (CD4>350 cells/µL) and TB were recruited under a larger phase 3 open label randomised controlled clinical trial. The CD45RO and CD62L markers were measured on CD4^+^ and CD8^+^ cells by flow cytometry. Samples were analysed at baseline, 3, 6, 12 months.

**Results:**

There was a significant increase of naive CD8^+^ cells (p = 0.003) and a decrease in effector CD8^+^ cells (p = 0.004) among participants in ART/TB treatment arm during the first 6 months. Withdrawing ART led to naive CD8^+^ cells reduction (p=0.02) to values close to baseline. An increase of naive CD8^+^ cells after 6 months of TB treatment in TB alone treatment arm (p=0.01) was observed. A trend towards increment of naive CD4^+^ sub sets in either treatment arms was observed.

**Conclusion:**

Interrupting ART alters CD8^+^ but not CD4^+^ sub-sets in patients with less advanced HIV infection and TB.

## Introduction

One in every four of human immunodeficiency virus type-1 (HIV-1) infected persons in the world is diagnosed with active pulmonary tuberculosis (TB)^1^. Tuberculosis accelerates progression of HIV infection to acquired immune deficiency syndrome (AIDS) compared to HIV patients without TB^2^,^3^. In Uganda at least 39% of TB cases in adults are complicated with HIV infection which poses a risk of high fatality rates^4^.

The HIV infection is characterised by progressive depletion of not only naive and memory CD4^+^ T cell sub-sets, but also the naive subset of CD8^+^ T cells from peripheral blood, lymphoid organs and mucosal tissues leading to immunodeficiency^5^,^6^. On the other hand, TB leads to increased immune activation, loss of both naive and MTB specific CD4^+^ T cells by apoptosis and defective cytokine production^7^,^8^,^9^. Although TB treatment alone has shown no significant changes in viral load, CD4^+^ and CD8^+^ T cell sub-sets, ART causes reduction in viral load and immune activation, restoration of naive T cells in HIV/TB patients^10^,^11^,^12^.

Much as interrupting ART in HIV/TB patients has its benefits like relief from tablet burden, reduction in costs and side effects of drugs on the patient, it poses risks such as emergence of drug resistance, resumption of HIV-induced immune suppression and chronic inflammation that may be detrimental^13^,^14^. The effect of interrupted ART on the reconstitution of naive and memory T cells during TB treatment in HIV/TB patients is not well understood. Other studies have reported that interrupted ART was as effective as continuous ART^15^. Therefore, in this report, we were answering a question of whether interruption of ART regimen during HIV/TB co-infection distorts the balance of naive and memory T cell reconstitution. To answer this question, HIV infected Ugandan adults with active pulmonary TB with CD4 > 350 cells/ml at baseline, 3,6 and 12 months were examined. These participants were part of randomised controlled clinical trial and were treated with both a combination of TB treatment and a 6-month punctuated course of ART (n=39) or TB treatment alone (n=37). Using flow cytometry, we measured changes in naive, memory and effector markers on T cells during the 6 months while ART was administered and when ART was withdrawn.

## Methods

### Participants

This is a very important study which was nested in a larger phase 3 clinical randomised study entitled “ Randomised Clinical Trial of 6 month punctuated course of antiretroviral Therapy (PART)- ClinicalTrials.gov identifier: NCT007847. Participants were Ugandan HIV+ adults with active pulmonary TB and CD4 > 350cells/µL. In the current study, the authors sought to establish “the effect of interrupted anti-retroviral treatment on the reconstitution of memory and naive T cells during tuberculosis treatment in HIV patients with active pulmonary tuberculosis”. It is very well written with no major ethical issues.

In the large study, participants who were HIV positive with active pulmonary TB were recruited. The participants were randomised to receive 6 months of TB treatment alone in one treatment arm and the other treatment arm had 6 months of both ART and TB treatment. Participants aged between 18 and 60 years were recruited from Uganda-Case Western Reserve University Research Collaboration (UCWRU) within Mulago Hospital Complex in Kampala, Uganda and were followed for one year. Participants with HIV-1 infection and ART naive with CD4 count > 350cells/mm^3^ were eligible to participate in the study. Active pulmonary TB was confirmed with positive smear and /or culture results and HIV-1 was diagnosed by ELISA plus Western blot and confirmed with HIV RNA copy levels. Before enrolment participants signed informed consent. The study protocol was approved by the institution Review Board of Case Western Reserve University/University Hospitals, University of California at San Francisco, the Uganda National Council for Science and Technology and Joint Clinical Research Centre in Kampala, Uganda.

In the large clinical trial, two weeks after study enrolment, participants were randomised to receive either 6 months of standard tuberculosis treatment alone (treatment arm 1) or tuberculosis treatment plus a punctuated course of 6 months of ART (treatment arm 2) which included trizivir, and a combination of lamivudine, abacavir and zidovudine. For TB therapy, participants were initially treated with a standard regimen of directly observed TB therapy which involved two months of rifampin, ethambutol, isoniazid and pyrazinamide and then followed with four months of isoniazid and rifampin. Cotrimoxazole was provided daily to participants after they had completed 2 months of TB therapy or HIV-TB therapy.

### Measurements

#### Demographic and clinical data

Demographic data and clinical symptoms of tuberculosis were collected on standard forms. Participants then had a physical exam, baseline chest radiograph and sputum samples were collected for a smear microscopy and culture at enrolment. At two and five months after initiation of tuberculosis treatment, other sputum samples for Acid Fast Bacilli(AFB) smear and culture were obtained. The HIV-1 infection was diagnosed by means of ELISA and Western blot test and confirmed with HIV RNA copy levels.

#### Flow cytometric analysis of markers of memory, naive and effector T cell populations

**Figure 1:**
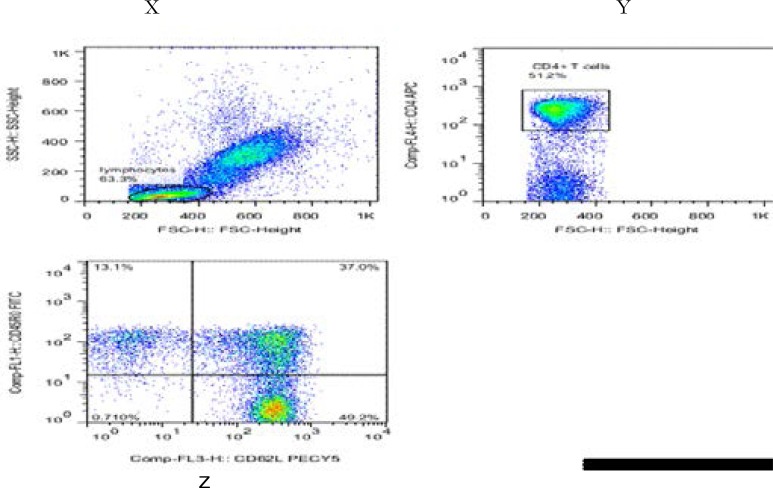
How acquired flow cytometric data was analyzed to define naive, memory and effector markers on CD4+ and CD8+ T cells

Immunophenotyping was performed. For flow cytometry the following antibodies were used in two panels: the first panel included anti-CD4 allophycocyananin (APC), anti-CD62L phycoerythrin Cy5 (PE Cy5), and anti-CD45RO fluorescein isothiocyanate (FITC), the second panel had anti-CD8 APC, anti-CD62L phycoerythrin Cy5 (PE Cy5), and anti-CD45RO fluorescein isothiocyanate (FITC). Mouse monoclonal isotypic control conjugated with PE, PE Cy5, FITC, and APC were used to determine non-specific binding and to set gating boundaries. All antibodies were obtained from Becton Dickson (BD) Pharmingen. Cells were acquired by a FACS Calibur flow cytometer (BD Bioscience) using Cellquest software (BD Bioscience) with further analysis performed using FlowJo Software (Tree Star, San Jose, CA). 25,000–50,000 cells were analyzed for each condition. Lymphocytes were gated based on forward and side scatter characteristics and then CD4^+^ and CD8^+^ T cell populations determined. Then presence or absence of CD45RO and CD62L were determined on CD4^+^ or CD8^+^ T cells (Figure 1).

Analysis was done on dot plots using Flowjo programme as follows: Lymphocytes were gated on forward and side scatter (dot plot **X)**. The CD4+ Tcells were gated on lymphocytes (dot plot **Y**). Dot plot **Z** illustrates expression of CD45RO and CD62L on CD4+ T cells. Likewise the same was done on CD8+ T cells. These proportions were then used to define naive (CD45RO-/CD62L+), memory (CD45RO+/CD62L+) and effector (CD45RO+/CD62L-) markers on CD4+ and CD8+ T cells.

#### Statistical considerations

Baseline characteristics plus comparison between treatment arms were analysed using non-parametric unpaired t test. Changes in memory, naive and effector markers on CD4^+^ and CD8^+^ T cell subsets at 0, 3, 6 and 12 months within each treatment arm were evaluated using Wilcoxon matched pairs test. This analysis was completed with Prism software version 4.0 (San Diego California USA). A p-value of < 0.05 was considered to show statistical significance.

## Results

Seventy six HIV-1 infected participants of whom thirty nine received ART with concurrent TB treatment and 37 received TB treatment alone, were analysed. Demographic baseline characteristics of the two groups did not significantly differ from each other (Table 1).

**Table 1:** Demographic and baseline characteristics of study participants. Median values and interquartile ranges of baseline characteristics were calculated. P values were calculated for the different variables between the groups.

Characteristic	TB treatment alone n=37	ART/TB treatment n=39	P
Age median (range) years	33 (19–54)	32 (23 –49)	.44
Male sex (%)	24 (65)	23 (59)	.98
CD4 absolute cell count median (range) cells/ µL	609.5 (374–1368)	532.5(283–1415)	.31
Viral load median (range) log_10_ copies/ml	4.4 (2.0–5.9)	4.4 (2.0–5.9)	.48

The absolute CD4 T cell counts did not differ statistically between the different study visits within each treatment arm (Table 2).

**Table 2:** Absolute CD4 cell count analysis between the study visits in the two arms

Study visit	Tuberculosis treatment alone	ART and Tuberculosis treatment
Baseline	609.5 (374 – 1368)	532.5 (283 – 1415)
6 months	527 (225 – 1331)	593 (348 – 2088)
12 months	609 (209 – 1252)	527 (291 – 1207)

* values in median (range)

### Punctuated ART combined with TB treatment alter CD8^+^ T cell subsets

**Figure 2:**
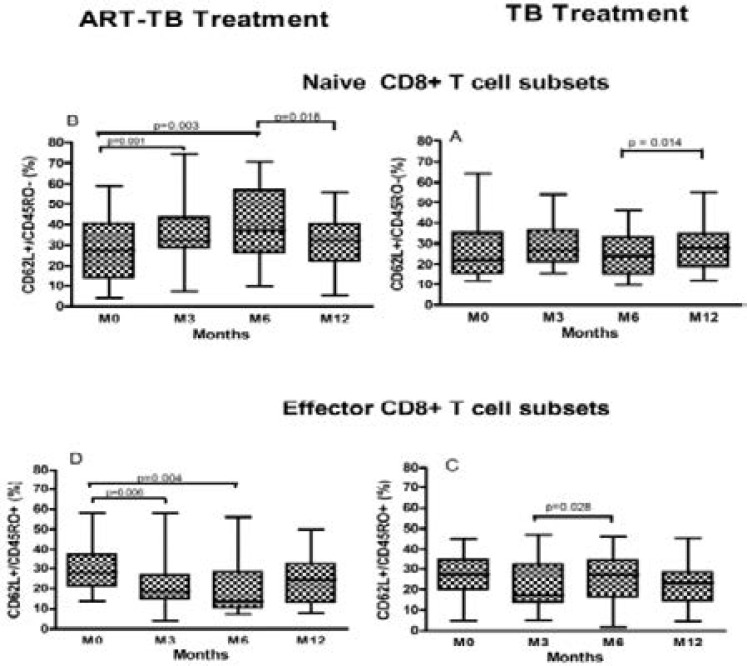
Changes in median of CD8+ cell subsets were measured in participants who received HIV-TB treatment (B & D) and those on TB treatment alone (A & B). Percentage expression of naive (CD62+/CD45RO-; A, B), effector memory (CD62L+/CD45RO+; C, D) markers was analysed from baseline, 3, 6 and 12 months. Boxes indicate the interquartile ranges; whiskers indicate the highest and lowest values while the horizontal lines transecting the boxes indicate the medians. Wilcoxon matched pairs test were performed to compare the median changes in these CD8+ T cell subsets within each treatment arm.

Proportions of naive (CD62L+/CD45RO-), memory (CD62L+/CD45RO+) and effector (CD45RO+/CD62L-) CD8^+^ and CD4^+^ T cell subsets were measured at enrolment and every 3 months for a period of one year. There was a significant increase of naive CD8^+^ T cells during the 6 month period of combined ART/TB treatment (p=0.003) and a significant decrease in effector CD8^+^ T cells (p=0.004, figure 2, graph B and D respectively). However, when ART treatment was withdrawn, naive CD8^+^ T cells reduced significantly (p=0.02). Memory CD8^+^ T cell proportions remained relatively stable in both treatment arms. During 6 months, naive CD8^+^ T cell sub-sets increased significantly among participants in ART-TB treatment arm than in the TB treatment arm (p=0.002, table 3). After completion of 6 months of TB treatment, the naive CD8^+^ T cells increased significantly in the TB treatment alone arm (p=0.01, figure 2, graph A). During the first 3 months of TB treatment in the TB treatment alone arm, effector CD8^+^ T cells decreased. Although this decrease was not significant, the next 3 months of TB treatment effector CD8^+^ T cells increased significantly to baseline levels (p=0.03, fig.2, graph C).

**Table 3:** Changes in CD8^+^ and CD4^+^ T cell subsets in both treatment arms

T cell subset	CD8^+^		CD4^+^	
	TB	ART/TB	TB	ART/TB
**Naive, % (IQR)**				

Baseline	22.0(15.5–35.0)	27.6(14.2–40.6)	48.0 (43.0–54.0)	45.0(35.6–58.5)
3 months	26.9(21.0–36.5)	32.1(28.8 –43.5)	49.0(40.5–55.0)	49.0(40.0–62.0)
6 months	24.1(15.3–33.1)	37.1(26.1–57.2)	48.0(39.0–54.0)	48.5(37.0–60.5)
12 months	28.0(18.9–34.8)	32.1(22.1–40.4)	49.5(39.5–58.5)	49.0(37.0–62.0)

**Memory, %** **(IQR)**				

Baseline	11.1(5.4–16.4)	12.7(9.6–16.7)	31.3(23.0–43.8)	27.5(20.4–37.5)
3 months	9.2(7.2–18.9)	11.9(8.7–17.1)	33.7(29.1–36.8)	30.5(22.3–39.4)
6 months	12.7(8.4–22.1)	12.6(6.9–17.3)	34.5(30.5–43.8)	32.8(22.5–39.4)
12 months	12.1(8.9–19.2)	13.3(6.9–19.1)	31.2(25.9–41.2)	32.7(22.3–38.2)
**Effector, %** **(IQR)**				

Baseline	27.3(19.7–35.0)	27.9(21.1–37.5)	10.7(7.5–14.8)	9.5(6.2–18.8)
3 months	17.2(13.4–32.1)	18.6(14.4–26.7)	11.4(9.9–16.3)	10.4(6.8–18.4)
6 months	27.0 (16.3–34.8)	13.6(10.3–28.2)	10.4(9.3–13.5)	12.1(5.9–17.7)
12 months	23.3(14.3–28.3)	24.5(12.9–32.6)	7.5(4.9–15.5)	10.8(6.6–18.6)

Note: IQR; Interquartile Range

Nonparametric unpaired t-test was used to compare changes between the two treatment arms.

Wilcoxon matched pairs test was used to compare changes within each treatment arm.

^α^ p<0.05 was statistically significant of comparison across treatment arms

### CD4^+^ T cell subsets were not altered by combined HIV-TB treatment

There were no significant changes in the proportions of CD4^+^ T cell sub-sets in combined HIV-TB treatment as well as TB treatment alone throughout the 12 months of study. However, the naive CD4^+^ T cell sub-sets showed a trend towards increment in both arms but these changes were not significant (HIV-TB treatment: naive CD4^+^ T cell sub-sets, between baseline and 6 months p= 0.66; TB treatment alone: p=0.98, table 3).

## Discussion

In the majority of chronically HIV-infected individuals left untreated, depletion of CD4^+^ T cells, changes in distribution and function of T cell sub-populations and alterations in CD4:CD8 T cell ratios are some of the consequences of uncontrolled viral replication. This is made worse in HIV/TB co-infected patients with TB driving immune activation which enhances viral replication. Upon administration of effective anti-retroviral therapy and TB treatment in HIV-TB patients, the peripheral T cells increase and alterations of some T cell sub-populations occur^16^,^17^. In this prospective cohort study of the effect of interrupted ART on T cell sub-sets during treatment of active pulmonary tuberculosis among HIV infected patients with CD4^+^ cell counts of > 350 cells/µL, we observed that ART administered concurrently with TB treatment causes a significant increase in naive CD8^+^ T cells. The ART intervention increased the proportion of naive CD8^+^ T cells during 6 months of treatment and this proportion declined close to baseline values when ART was withdrawn. We also report that during the 6 months of intervention, the effector memory CD8^+^ T cells decreased significantly. In the TB treatment alone arm, after 6 months of treatment, we observed a significant increase in naive CD8^+^ T cells. T cell sub-population reconstitution has been a subject of interest in HIV and TB infections for long time in research; however, most studies have been limited by focusing on either HIV or TB infection^17^,^18^,^19^. This study is one of its kind that focuses on dual infections.

CD8^+^ T cells play an important role of killing cells that harbour intracellular pathogens either through secreting cytokines like interferon gamma or production of lysing biomolecules like granulysin/perforin. Loss of naive T cells and skewing of T cell sub-populations in HIV infection has been associated with rapid HIV disease progression^18^. In this study, the patients were in a less advanced stage of HIV which provided the benefit of rapid increase in the naive CD8^+^ T cell sub-population. This means that HIV and TB had not done tremendous damage to CD8^+^ stromal naive T cells to hinder their expansion. Tuberculosis is well known to rapidly increase HIV disease progression through facilitating T cell activation^3^,^5^,^7^. Additionally, T cell activation is known to be one of the contributing forces that hinders naive T cell regain and expansion. Several studies including this particular one have shown that tuberculosis treatment in HIV-TB patients reduces activation markers on CD4^+^ and CD8^+^ T cells in HIV patients co-infected with tuberculosis^10^,^17^,^21^. Changes in homeostatic mechanisms and markers could have played a role of reducing circulating numbers of effector CD8^+^ T cells in the periphery to antigen reservoirs and primary sites of infections. It could also suggest that homeostatic micro-environment that is important in maintenance of mature T cells had been partially damaged/or exhausted by HIV infection. Despite viral suppression to very low levels, it seems low levels of HIV antigen production persists in the tissue reservoirs. This is evident in that, the moment ART was withdrawn, naive CD8^+^ T cells fell to levels close to baseline. It is worthwhile to note that when naive CD8^+^ T cells reduced, there was no significant increase in the memory CD8^+^ T cell sub-populations. This could suggest that damage was already done to naive CD8^+^ T cell sub-population at an earliest stage of uncontrolled HIV replication and their expansion was already impaired prior to initiation of treatment. Then the importance of early initiation of ART on recovery of naive T cell pool remains to be seen. The sudden decrease at 3 months in effector CD8^+^ T cells seen TB treatment arm could be due to introduction of treatment as seen elsewhere^20^.

It has been shown that ART improves restoration of naive CD4 T cell counts in HIV infected individuals^9^, ^11^,^20^. Some researchers demonstrate that TB treatment increases CD4^+^ T cell counts in HIV-TB co-infected persons^17^ while others show no change during therapy^15^.We report no significant changes in both absolute CD4^+^ counts and T cell sub-set proportions in both treatment arms. This observation has been supported by studies that have done proliferation experiments which have demonstrated that CD4^+^ proliferation is driven by CD4^+^ cell depletion and viral load while CD8^+^ cell proliferation is driven by viral load alone. Since these patients were at a less advanced stage (CD4 T cell counts >350cells/µL) of disease progression, when ART was interrupted there were no significant changes in naive, memory and effector CD4^+^ T cell proportions in HIV/TB treatment group. This has also been noted in some studies there is limited influence on the magnitude of change of naive and memory CD4^+^ T sub-sets in asymptomatic compared to advanced stage of disease^18^,^20^. In contrast to advanced HIV patients, previous studies have shown an early increase of memory and late increase of naive CD4^+^ cells while on ART^20^. This suggests that gradual increase of these sub-sets is dependent on the pre-existing naive T cells in HIV infected patients. If this remains the case for HIV patients co-infected with TB remains a question to be answered.

## Conclusion

Our analysis in this study provides more insight in the reconstitution of T cell sub-populations in HIV-TB infected patients with high CD4^+^ T cell counts. Although initially, this study was not designed with a systematic ART interruption model, interruption of ART did not yield immunological benefits which suggest poor clinical outcomes to the patients.

One weak point of this study is that there was no control group to compare with the percentages of T cell sub-sets in the two treatment groups. For ethical reasons such a study would never be conducted. Further research needs to be performed in functional analysis of these phenotypes to characterise the quality of these cells during-HIV/TB co-infections.
